# Case Report: An Application of Wellbeing Science for the Development of Adolescent High-Performance Athletes in the Australian Football League

**DOI:** 10.3389/fpsyg.2022.856241

**Published:** 2022-06-10

**Authors:** Erin Hoare, Nicky Couston, Kate Hall

**Affiliations:** ^1^Australian Football League, Melbourne, VIC, Australia; ^2^IMPACT - the Institute for Mental and Physical Health and Clinical Translation, School of Medicine, Barwon Health, Deakin University, Geelong, VIC, Australia; ^3^School of Psychology, Deakin University, Geelong, VIC, Australia

**Keywords:** mental wellbeing, young high-performance sport, elite sport, youth athletes, community case studies, Australia

## Abstract

Elite athletes experience both universal and sport-related mental health risks. Young high-performance athletes on pathways to professional sport also face the additional challenges associated with the developmental period of adolescence and early adulthood, making prevention and mental health promotion critical in this population group. This community case study considers the wider youth mental wellbeing evidence base, alongside primary prevention in elite sport, and proposes a model of wellbeing for the specific implementation in youth high performance athletes in the Australian setting. The Mental Fitness Model is based on the PERMA theory of wellbeing, which comprises positive emotion, engagement, relationships, meaning, and accomplishment, and is tailored specifically to the unique needs of young high-performance athletes in Australia. The Model sits within a host of evidence-based, appropriately resourced, wellbeing science activities, coordinated by an overall strategy that allows monitoring and continuous improvement. As such, we propose this application of wellbeing science is highly novel for the youth high performance setting. Future work is needed to test the feasibility of this model in an applied context. Further work is also needed to integrate specifically cultural considerations for wellbeing, and to integrate the lived experiences of young people through participatory research. This model is proposed to hold unique promise to meet the mental wellbeing needs of young high-performance athletes, whilst promoting positive mental health that can track into adulthood.

## Introduction

High performance sport is increasingly recognized as a unique setting which poses risks to mental wellbeing. Athletes experience universal risks, such as genetic pre-disposition, stressful life circumstances, and relationship difficulties, in addition to sport-specific risks such as performance pressures, social media and other public scrutiny, and physical injury risks (Purcell et al., 2019). Meta-analytic research suggests comparable prevalence of common mental disorders (i.e., depression and anxiety) between athletes and the general population (Gouttebarge et al., 2019). There is, however, increasing recognition of the high-performance environment specific stressors that can contribute to the onset and course of mental ill health, and therefore form potential targets for prevention and early intervention (Currie et al., 2021).

Mental health researchers are largely in agreement that an athlete's mental health needs are paramount to physical wellbeing, and that it is both physical and mental wellbeing that are likely to contribute to optimizing athletic performance (Rice et al., 2016; Reardon et al., 2019). Whilst the treatment and management of athlete mental ill health is fundamental to overall athlete wellbeing, it is now recognized that promoting mental wellbeing serves a role beyond offsetting risks of mental disorders (Uphill et al., 2016). Mental wellbeing, and positive psychology more broadly, shifts focus from pathology and deficit focused paradigms of mental health, toward the conditions that support an individual to flourish (Seligman and Csikszentmihalyi, 2014). In other words, mental health is considered more than the absence of mental ill health but encapsulates a global state of wellbeing that focuses on a holistic approach to overall health and human development such that individuals are able to live meaningfully and positively contribute to society.

A large proportion of the mental wellbeing literature to date has focussed on young people, reflective of the known optimal age period for prevention in early life (Arango et al., 2018). In particular, the adolescent age period of 13–18 years comprises a period of rapid developmental, social and emotional changes including increased independence, autonomy, physical maturation, and also a known period of increased risk behaviors (Sawyer et al., 2018). Young people engaged in high performance sports experience the universal risks during this age period, alongside sport specific experiences such as increased pressure to perform, physically and mentally demanding training programs, and potential alternative education programs (Brenner et al., 2019).

Given the known benefits of primary prevention in this age group, alongside the growing understanding of nurturing mental wellbeing among athletes, there exists a unique opportunity to develop and implement a model of wellbeing for young high-performance athletes. This community case study reports on the relevant literature, identifies elements of mental wellbeing most pertinent to this group informed by positive psychology, and describes the application of a model of wellbeing for the high-performance space among young Australian athletes. It is envisaged that such a model could be adapted to meet the needs of young high-performance athletes across other codes and settings, as well as remaining iterative to the emerging evidence body among this specific population group.

## Context

The Australian Football League (AFL) is the pre-eminent, professional sporting competition of Australian rules football and encompasses both men's and women's (AFLW) professional leagues. The AFL is the governing body for the sport at the professional level, and importantly holds responsibility for the participation and talent development infrastructure that supports the professional competition. The AFL's National Talent Pathway is the AFL youth program for talent identified players aged 16–19 years in high performance leagues and Academies throughout the country. There are approximately 1,500 athletes identified annually in the program who are invited to engage in intensive programs to prepare for possible drafting to the national leagues. These identified young high performance athletes are required to engage in training, matches and activities to develop their talent and ability to perform under pressure, whilst being scrutinized by AFL Clubs for their suitability to become an AFL/AFLW player. Providing further context for this current work is the AFL Industry Mental Health Strategy, which was launched in 2019 covering period 2020–2022 (https://resources.afl.com.au/afl/document/2020/12/16/49fbf87a-7290-4a88-a0a3-5c98c79e6e4a/AFL-Mental-Health-Wellbeing-Strategy_2020_2022.pdf). The aligned Mental Health Strategy guides an industry wide approach to mental health and wellbeing and includes all associated activities across the industry including those within the National Talent Pathway. The AFL Mental Health and Wellbeing in the National Talent Pathway position statement further contextualizes this current work (https://resources.afl.com.au/afl/document/2021/11/04/67edea0c-d0fc-4fdb-ae33-1e0a86b1e562/Talent-Pathway-Wellbeing-Position-Statement.pdf?_ga=2.22362163.949308775.1651457423-1818937647.1629779017).

## Programmatic Elements

### Literature Review

A review of literature was conducted to identify applications of mental wellbeing in young high-performance sport. The Scale for the Assessment of Narrative Review Articles informed the methods for this review (Baethge et al., 2019). Whilst the articles selected for review were based on their relevance to the context of this specific study, it is possible that some literature was overlooked and thus this approach does not assume to be exhaustive.

### Mental Wellbeing

The reviewed literature suggests that there is consensus that positive mental health is not exclusively the absence of psychopathology, and equally, experiencing low levels of wellbeing does not confirm a diagnosable mental health condition. Mental wellbeing, however, is not universally defined, although it is generally accepted that wellbeing is best described as a multi-dimensional construct, that incorporates pleasure and happiness, as well as psychological and other forms of functioning, and ultimately concerns the factors that allow optimal human functioning (Dodge et al., 2012; van Agteren et al., 2021). Eudemonic (i.e., personal fulfillment and living one's values) and hedonic (i.e., the pursuit of happiness and other pleasurable feelings) theories have characterized mental wellbeing definitions historically, however such definitions have been criticized for focusing exclusively on positive feelings and positive functioning which can be restrictive and specific to the culture in which individuals live (Disabato et al., 2016; Huta, 2017). Further, alongside the lack of consensus for theoretical underpinnings of mental wellbeing, there are also consequential inconsistencies in the measurement of mental wellbeing which further complicate the field.

### Mental Wellbeing in Young People

The developmental perspective requires a model of mental wellbeing that applies to young people, of which positive psychology appears to hold promise based on the exponential growth of literature in this field aimed at adolescence and school settings generally (i.e., positive education, Seligman and Adler, 2018). Martin Seligman, a pioneer in positive psychology, proposed that wellbeing is comprised of five elements being positive emotions, engagement, relationships, meaning and accomplishment, which he refers to as the PERMA model of wellbeing (Seligman, 2012; Seligman and Csikszentmihalyi, 2014). Positive emotion refers to happiness, joy, love, compassion, and other emotions. Engagement refers to being present in the current moment and engaging entirely in the task at hand. Positive relationships refers to interactions with others that reflect supportive, loving and valued relationships. Meaning refers to experiencing value, worth and holding a purpose. Accomplishment refers to working toward and achieving goals. Seligman proposed that the above factors are major contributors to wellbeing, and they comprise defined and independent constructs that are intrinsically motivating to an individual (Seligman, 2018).

Seligman's PERMA model has shown moderate associations with other subjective measures such as those that screen for depression, anxiety, and stress (Kern et al., 2015; Butler and Kern, 2016). A longitudinal study of adolescents demonstrated that indicators corresponding to the PERMA model successfully predicted educational attainment and civic activities such as volunteering. This suggests that promoting positive mental wellbeing in younger years may support some healthy developmental milestones in adulthood such as employment and community engagement (O'Connor et al., 2017). The uptake of the PERMA model in school settings in Positive Education reflects the perceived appropriateness and value of this approach for supporting young people amongst community members and stakeholders (Slemp et al., 2017; Waters et al., 2017).

A major criticism of the PERMA model, and positive psychology generally, is that it is overly individualistic and culture bound in terms of domains in focus for subjective wellbeing (Gruman et al., 2018). This is particularly important given the known role of social determinants in the development and maintenance of mental wellbeing across the lifespan (Allen et al., 2014). Such determinants may be cultural, political, historical, and/or social such as the impact of poverty, racial discrimination, exposure to stressful life events, and access to community resources. In the Australian setting, the historical oversight of the specific social, emotional, and cultural experiences of wellbeing among First Nations people universally across models of mental health and wellbeing cannot be overemphasized (Terare and Rawsthorne, 2020; Wilson and Waqanaviti, 2021). This was highlighted in the Australian Government Working Together: Aboriginal and Torres Strait Islander Mental Health and Wellbeing Principles and Practice body of work which aimed to provide appropriate resources to support mental health professionals who work with First Nations people experiencing mental health and wellbeing concerns (Purdie et al., 2010; Gee et al., 2014). It was noted that this work was in response to a dearth of literature to date in culturally appropriate resources that enable professionals to provide support in the context of suffering, grief and other forms of distress resulting from previous policies and practice for Aboriginal and Torres Strait Islander people. Gee and colleagues discussed understandings of social and emotional wellbeing from an Aboriginal and Torres Strait Islander perspective which includes centralizing culture alongside Aboriginal and Torres Strait Islander world views, and encompasses domains of health and wellbeing including connection to land or “country”, ancestry, kinship and community). This is important in the AFL context because of the long-standing centrality of the game to Indigenous communities throughout Australia (Gee et al., 2014; Gorman, 2017).

### Mental Wellbeing in Young High-Performance Athletes

The need to understand the uniqueness of mental health in the elite sporting domain is firmly established, as demonstrated through the recent publication of the International Olympic Committee consensus statement on mental health in elite athletes (Reardon et al., 2019). Importantly, Purcell and colleagues identified that whilst the International Olympic Committee's consensus statement provided welcomed recommendations for the treatment of mental illness among athletes, there was reduced attention to prevention and mental wellbeing promotion initiatives. Purcell et al. acknowledged that while mental health literacy and intervention models of care are prevailing frameworks in this burgeoning literature, primary prevention and wellbeing approaches have not been broadly examined (Purcell et al., 2019). In response to this, Purcell and colleagues proposed a framework for promoting mental health and wellbeing for elite athletes that comprises self-management skills among athletes, building capacity among stakeholders to support mental health, and emphasizing the need for multidisciplinary supporting teams (Purcell et al., 2019). Purcell et al. highlighted the need for whole systems of support that contribute to environments that promote and nurture mental health and that respond adequately to athletes' needs.

Whilst the above literature made important advances in relation to mental health, the area of wellbeing and positive mental health is in further infancy in the elite sport environment. Lundqvist et al. explored conceptual understandings of wellbeing in the elite athlete domain, finding that most wellbeing-based studies have used weak theoretical rationales or conceptual models of wellbeing, and failed to recognize the distinction between wellbeing in the general community and wellbeing among athlete groups (Lundqvist, 2011; Lundqvist and Andersson, 2021). Moreover, the identified literature on wellbeing among athletes has been limited by lack of use of sound, reliable and valid measures of wellbeing (Cooke et al., 2016; Giles et al., 2020). Despite limitations, the available literature to date has sought to identify the factors (and therefore, strategies) that can be utilized to protect and improve athlete wellbeing. Specifically evidence to date has sought to understand the lived sports environment whilst considering the individual, social and community factors that unique to athletes (e.g., athletic identity, media and other pressures, physical injury) (Lundqvist and Sandin, 2014; Küttel et al., 2020).

Whilst literature on the facets of mental health and wellbeing support for elite athletes are emerging, the experiences of young high performance athletes (i.e., under the age of 18 years) is in infancy (Xanthopoulos et al., 2020). The young athlete experiences developmental changes that characterize adolescence generally including physical maturation, social and peer group transitions, and a shift from dependent to increasingly independent relationships. The sport-specific factors that contribute to mental wellbeing include performance expectations, intensive training programs that may displace academic and social opportunities, and other pressures that characterize the high-performance domain. Whilst some literature historically demonstrated lower rates of mental illness among young high performance athletes, this has since been suggested to be explained, at least in part, by stigma associated with help seeking (Bauman, 2016). It is now understood the young athlete experiences mental ill health at similar rates to the general population (Gulliver et al., 2015; Rice et al., 2016).

In terms of mental wellbeing, there is very little research in terms of models of mental wellbeing designed specifically to support young high-performance athletes. Of the existing literature conducted among young high performance athletes, mental wellbeing has been measured as a secondary outcome, such as in studies designed to promote mental health and optimize performance through family-based intervention activities (Donohue et al., 2015), mindfulness based interventions (Longshore and Sachs, 2015), and for young high performance athletes identified at risk for mental health problems (Tester et al., 1999). Other research has sought to examine aspects of mental wellbeing, or trialed standalone positive psychology interventions such as practicing gratitude (Gabana, 2019). In terms of models of wellbeing for young high-performance athletes, we identified one example in the published literature which proposed, through theoretical review, the appropriateness of PERMA among Finnish junior ice hockey players (Uusiautti et al., 2017). Uusiautti et al. operationalised PERMA for elite junior ice hockey athletes and identified opportunities to support mental wellbeing in this group. Whilst this work offers unique insight into the theoretical appropriateness of PERMA, it does not propose a specific model of wellbeing and strategies for implementation.

### Summary of Literature

The above literature review highlights that the evidence to date relating to elite athlete mental health has primarily focussed on psychopathology and treatment of mental illness, with little focus on the mental health promoting factors and strategies to support mental wellbeing. We identified that there may be utility for positive psychology, and in particular the PERMA model, to foster positive wellbeing however the mental wellbeing literature to date does not include a universally accepted definition or optimal approach. This is particularly the case for young people, with growing evidence to support the use of positive education that broadly targets the domains of the PERMA model in the education setting for young people aged 18 years and younger (Slemp et al., 2017; Waters and Loton, 2019). Despite its recognized utility, the PERMA model is limited by its individualistic approach, and the lack of recognition of the cultural and community factors that interact to impact mental wellbeing (Kern et al., 2020). Considering the Australian setting, we identified an Aboriginal and Torres Strait Islander model of social and emotional wellbeing to incorporate the social determinants of mental wellbeing more appropriately. Finally, we identified that the understandings of mental health and wellbeing are growing for the adult athlete population, however, less is known among young high-performance athletes who experience the dual risks of developmental changes that accompany adolescence, and the expectations and challenges that characterize high level sport. To our knowledge, there has been one proposal which discussed how the PERMA model could be operationalised in the young high-performance space, however, there does not appear to be a current model of wellbeing that has been developed, implemented and evaluated in a real-world sport setting.

## The Mental Fitness Model

Given the above literature review and identified gaps in evidence to date, we sought to develop and propose a model of mental wellbeing for young high-performance athletes in the Australian Football League. Utilizing the above literature, combined with the investment within the AFL industry which recognizes the period of adolescence as critical to prevent and promote positive mental wellbeing that can track into adulthood, the Mental Fitness Model was developed. The purpose of this model was to enable a proactive and supportive culture of wellbeing in the National Talent Pathway, to provide environments that allow young high-performance athletes to develop and thrive, and to provide a framework which other junior sporting programs may adopt to support the wellbeing of their athletes.

**Figure 1:**
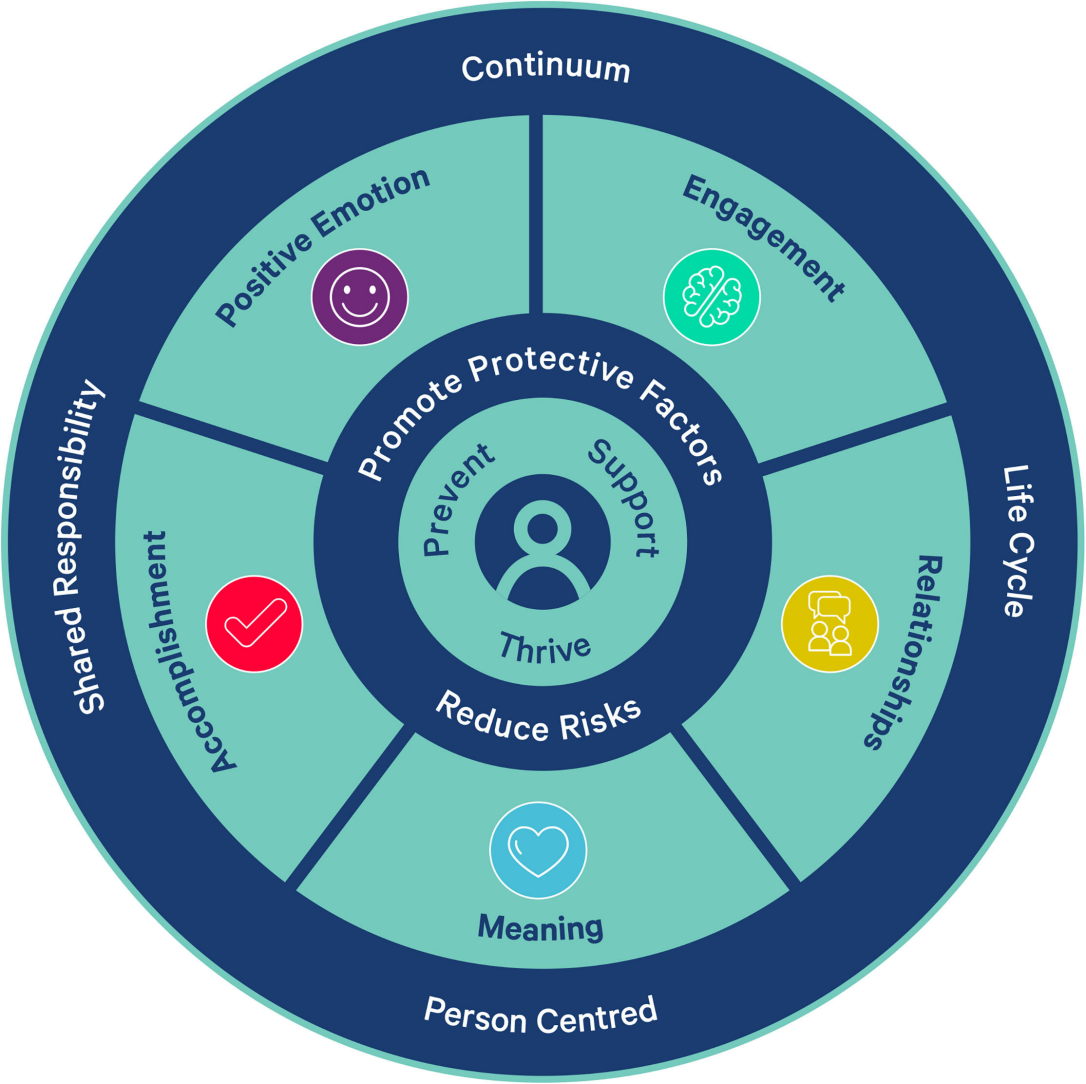
Mental fitness model.

The Mental Fitness Model (Figure 1) is informed by Seligman's PERMA model alongside several cross-cutting principles drawn from the AFL Industry Strategy which adopts the mental health continuum approach to mental wellbeing and ecological systems model for mental health (Keyes, 2002; Purcell et al., 2019). The model components, including supporting literature, proposed mechanisms, and example activities within the specific AFL context, are reported in Supplementary Table 1. The Mental Fitness Model focuses on the individual athlete at the center of the Model which considers the athlete's individual differences and their environment as interdependent contributors to wellbeing. This model acknowledges both the individual and the sporting system as both intervention targets. Three pillars of the mental health continuum are articulated in the model: prevent, support and thrive (flourish) (Keyes, 2002). The addition of mental fitness to the continuum was made in acknowledgment of the unique mental requirements of high-performance environments such as elite sporting contexts. The individual building blocks of individual athlete wellbeing corresponding to the PERMA model of positive emotion, engagement, relationships, meaning, and accomplishment were integrated into the model.

The following levels, informed by Purcell's comprehensive mental health framework (Purcell et al., 2019), incorporate the promotion of protective factors, and the reduction of risks that occur in the individual, family, team and social, and other environments. In practice this incorporates the AFL specific risk and protective factors faced by young people such as age of draft and subsequent changes to education, employment and training engagement, being an individual member of a larger team, Australian-centric nature of the sport, and the unique challenges of the men's and women's programs (e.g., the shortened season length in the women's program). The final level proposes the wider macro level processes that underpin the building blocks of wellbeing such as the National sporting environment, public and social media. The Mental Fitness model was developed to be informed by theoretical frameworks for wellbeing, psychiatry's staging model, public health models such as social determinants of health and health promotion approaches. There is a continuous improvement focus that sought to evaluate and revise in an iterative way, as the program evolved and new evidence emerged relating to mental wellbeing.

### Implementation

The above model guides the implementation of a host of wellbeing activities, all of which are designed to contribute to an environment of positive mental wellbeing. This aligns with earlier review of literature which highlights the need of moving beyond athlete awareness and encouraging help seeking, to creating mental wellbeing nurturing systems and environments (Purcell et al., 2019; Kern et al., 2020). A major component of this model is the delivery of the wellbeing curriculum to the young athletes. Whilst outside the scope of this paper in terms of program components (further work in relation to this program is forthcoming), briefly this curriculum focuses on building skills and strategies for young people to care for and build their own wellbeing. This reflects the core of the Model which is person centered in terms of individual skills development, as well as the interconnectedness of the environment in which they live. It also includes a parent education component to upskill parents and guardians in the wellbeing Model to build capacity in the home environment, and to create shared responsibility and language for young people's wellbeing. The delivery of wellbeing science is facilitated by AFL-employed, local wellbeing co-ordinators, who hold minimum or working toward Bachelor-level qualifications in health, education, or science. The young high-performance athletes are engaged in data surveillance regarding mental wellbeing, including the EPOCH measure which corresponds to the PERMA domains of wellbeing and adjusted for age-appropriateness (Kern et al., 2016).

It is important to note the implementation approach is designed based on best practice principles for enabling systems-level change (Braithwaite et al., 2018), and what has recently been promoted in the positive psychology literature (Kern et al., 2020). Specifically, the wellbeing activities are a set of strategic and coordinated activities, informed by the evidence-base as described above. This is in contrast to many wellbeing programs which can occur as standalone sessions with no overarching strategy or clearly defined evidence base on which they are developed. The role of wellbeing coordinators, who are credentialed in health, education or science, and who have completed professional development in wellbeing science and therefore hold appropriate skills and capacities, provide local, targeted and consistent supports to the young people within their settings. It is widely reported that to enable any positive health support for young people, there must be local leadership within the settings and communities in which the young people exist, that allow relationships which are enduring, localized and integrated (Bischoff et al., 2017; Hoare et al., 2019). Such personnel hold professional qualifications, and also hold community-specific knowledge of the players with whom they work. Lastly, on-going quality assurance and evaluation is achieved through surveillance and monitoring using reliable and validated measures of wellbeing. Whilst many wellbeing programs and initiatives are often developed and implemented, rarely do monitoring of outcomes and subsequent improvement of program components occur.

## Discussion

Our review demonstrated the need to consider the application of mental wellbeing science specifically for the young high performance athlete population. Whilst mental health treatment and coordinated systems of care are increasingly understood to be a critical aspect of high-performance sport generally, fewer literature has focussed on promoting mental wellbeing. Even fewer research studies have sought to explore models of wellbeing for the young high-performance athlete. Given what is known in regard to prevention during early years, the opportunities for early intervention leading to improved outcomes that can track into adulthood, and the uniqueness of elite sport specific experiences, there is strong rationale to explore how best to enable mental wellbeing in young athletes. The literature reviewed in this work led to the development of the Mental Fitness Model, which is based on the PERMA model of wellbeing, and adapted to the sport specific environment of the young Australian Footballer in the National Talent Pathway. This model sits within a suite of wellbeing activities that we propose are highly novel in terms of evidenced-informed program content, within-community resources (i.e., dedicated local wellbeing co-ordinators), and data surveillance to inform quality improvement and respond to real-time needs of athletes.

## Limitations

A limitation of the model to date is the need to further integrate specific cultural factors into conceptual framework and practice of mental wellbeing, such as that proposed in the Australian Government Working Together: Aboriginal and Torres Strait Islander Mental Health and Wellbeing Principles and Practice. We also identify the need for young athletes lived experiences to be incorporated into this work, including consideration toward language that could best support young athletes to conceptualize wellbeing. Lastly, our model was developed and implemented for the Australian Rules Football context, but we envisage this model, and in particular the above reported literature review, theoretical and practical considerations, to be applicable across sporting codes and settings.

## Author's Note

This case study was conducted as part of ongoing continuous improvement framework for the wellbeing program.

## Data Availability Statement

The original contributions presented in the study are included in the article/Supplementary Material, further inquiries can be directed to the corresponding author/s.

## Author Contributions

EH led the drafting of the manuscript, collated input from co-authors, and prepared the piece for submission. NC led the design, development, and delivery of the Model. KH oversaw all aspects of the Model development, the work prepared and reported in this manuscript, and provided senior expertise in psychology and wellbeing science. All authors contributed to the article and approved the submitted version.

## Conflict of Interest

All authors are employed by the Australian Football League.

## Publisher's Note

All claims expressed in this article are solely those of the authors and do not necessarily represent those of their affiliated organizations, or those of the publisher, the editors and the reviewers. Any product that may be evaluated in this article, or claim that may be made by its manufacturer, is not guaranteed or endorsed by the publisher.

## References

[B1] Allen J. Balfour R. Bell R. Marmot M. (2014). Social determinants of mental health. Int. Rev. Psychiatry. 26, 392–407. 10.3109/09540261.2014.928270 25137105

[B2] Arango C. Díaz-Caneja C. M. McGorry P. D. Rapoport J. Sommer I. E. Vorstman J. A.. (2018). Preventive strategies for mental health. Lancet Psychiat. 5, 591–604. 10.1016/S2215-0366(18)30057-9 29773478

[B3] Baethge C. Goldbeck-Wood S. Mertens S. (2019). SANRA—a scale for the quality assessment of narrative review articles. Res. Integr. Peer Rev. 4, 1–7. 10.1186/s41073-019-0064-8 30962953 PMC6434870

[B4] Bauman N. J. (2016). The stigma of mental health in athletes: are mental toughness and mental health seen as contradictory in elite sport? Br. J. Sports Med. 50, 135–6. 10.1136/bjsports-2015-095570 26626270

[B5] Bischoff R. J. Springer P. R. Taylor N. (2017). Global mental health in action: Reducing disparities one community at a time. J. Marital Family Ther. 43, 276–290. 10.1111/jmft.12202 27859402

[B6] Braithwaite J. Churruca K. Long J. C. Ellis L. A. Herkes J. (2018). When complexity science meets implementation science: a theoretical and empirical analysis of systems change. BMC Med. 16, 1–14. 10.1186/s12916-018-1057-z 29706132 PMC5925847

[B7] Brenner J. S. LaBotz M. Sugimoto D. Stracciolini A. (2019). The psychosocial implications of sport specialization in pediatric athletes. J. Athl. Train. 54, 1021–1029. 10.4085/1062-6050-394-18 31532693 PMC6805069

[B8] Butler J. Kern M. (2016). The PERMA-Profiler: a brief multidimensional measure of flourishing. Int. J. Wellbeing 6. 10.5502/ijw.v6i3.526

[B9] Cooke P. J. Melchert T. P. Connor K. (2016). Measuring wellbeing: a review of instruments. Couns. Psychol. 44, 730–757. 10.1177/0011000016633507

[B10] Currie A. Blauwet C. Bindra A. Budgett R. Campriani N. Hainline B.. (2021). Athlete mental health: future directions. Br. J. Sports Med. 55, 1243–4. 10.1136/bjsports-2021-104443 34344708

[B11] Disabato D. J. Goodman F. R. Kashdan T. B. Short J. L. Jarden A. J. P.. a. (2016). Different types of wellbeing? A cross-cultural examination of hedonic and eudaimonic wellbeing. Psychol. Assess. 28, 471. 10.1037/pas0000209 26348031

[B12] Dodge R. Daly A. P. Huyton J. Sanders L. (2012). The challenge of defining wellbeing. Int. J. Wellbeing 2, 222–235. 10.5502/ijw.v2i3.4

[B13] Donohue B. Chow G. M. Pitts M. Loughran T. Schubert K. N. Gavrilova Y.. (2015). Piloting a family-supported approach to concurrently optimize mental health and sport performance in athletes. Clin. Case Stud. 14, 159–177. 10.1177/1534650114548311

[B14] Gabana N. T. (2019). Gratitude in Sport: Positive Psychology for Athletes and Implications for Mental Health, Wellbeing, and Performance, in Theoretical Approaches to Multi-Cultural Positive Psychological Interventions. Springer International Publishing p. 345–370. 10.1007/978-3-030-20583-6_15

[B15] Gee G. Dudgeon P. Schultz C. Hart A. Kelly K. (2014). Aboriginal and Torres Strait Islander social and emotional wellbeing. 2, 55–68.

[B16] Giles S. Fletcher D. Arnold R. Ashfield A. Harrison J. J. S. M. (2020). Measuring wellbeing in sport performers: where are we now and how do we progress? Sports Med. 50, 1255–1270. 10.1007/s40279-020-01274-z 32103451 PMC7305091

[B17] Gorman S. (2017). Indigenous Past Player Forum Report.

[B18] Gouttebarge V. Castaldelli-Maia J. M. Gorczynski P. Hainline B. Hitchcock M. E. Kerkhoffs G. M.. (2019). Occurrence of mental health symptoms and disorders in current and former elite athletes: a systematic review and meta-analysis. Br. J. Sports Med. 53, 700–706. 10.1136/bjsports-2019-100671 31097451 PMC6579497

[B19] Gruman J. A. Lumley M. N. González-Morales M. (2018). Incorporating balance: challenges and opportunities for positive psychology. Can. Psychol. 59, 54. 10.1037/cap0000109

[B20] Gulliver A. Griffiths K. M. Mackinnon A. Batterham P. J. Stanimirovic R. (2015). The mental health of Australian elite athletes. J. Sci Med. Sport. 18, 255–261. 10.1016/j.jsams.2014.04.006 24882147

[B21] Hoare E. Thorisdóttir I. E. Kristjansson A. L. Sigfusdóttir I. D. Hayward J. Allender S.. (2019). Lessons from Iceland: developing scalable and sustainable community approaches for the prevention of mental disorders in young Australians. Mental Health Preven. 15, 200166. 10.1016/j.mhp.2019.200166

[B22] Huta V. (2017). An overview of hedonic and eudaimonic wellbeing concepts.

[B23] Kern M. L. Benson L. Steinberg E. A. Steinberg L. (2016). The EPOCH measure of adolescent wellbeing. Psychol. Assess. 28, 586. 10.1037/pas0000201 26302102

[B24] Kern M. L. Waters L. E. Adler A. White M. (2015). A multidimensional approach to measuring wellbeing in students: application of the PERMA framework. J. Posit. Psychol. 10, 262–271. 10.1080/17439760.2014.936962 25745508 PMC4337659

[B25] Kern M. L. Williams P. Spong C. Colla R. Sharma K. Downie A.. (2020). Systems informed positive psychology. J. Posit. Psychol. 15, 705–715. 10.1080/17439760.2019.1639799

[B26] Keyes C. (2002). The mental health continuum: from languishing to flourishing in life. J. Health Soc. Behav. 43, 207–222. 10.2307/3090197 12096700

[B27] Küttel A. Larsen C. Psychology E. (2020). Risk and protective factors for mental health in elite athletes: a scoping review. Int. Rev. Sport Exerc. Psychol. 13, 231–265. 10.1080/1750984X.2019.1689574

[B28] Longshore K. Sachs M. (2015). Mindfulness training for coaches: A mixed-method exploratory study. J. Clin. Sport Psychol. 9, 116–137. 10.1123/jcsp.2014-0038

[B29] Lundqvist C. (2011). Wellbeing in competitive sports—The feel-good factor? A review of conceptual considerations of wellbeing. Int. Rev. Sport Exer. Psychol. 4, 109–127. 10.1080/1750984X.2011.584067

[B30] Lundqvist C. Andersson G. (2021). Let's talk about mental health and mental disorders in elite sports: a narrative review of theoretical perspectives. Front. Psychol. 29:700829. 10.3389/fpsyg.2021.700829 34267715 PMC8275956

[B31] Lundqvist C. Sandin F. (2014). Wellbeing in elite sport: dimensions of hedonic and eudaimonic wellbeing among elite orienteers. Sport Psychol. 28, 245–254. 10.1123/tsp.2013-0024

[B32] O'Connor M. Sanson A. V. Toumbourou J. W. Norrish J. Olsson C. (2017). Does positive mental health in adolescence longitudinally predict healthy transitions in young adulthood? J. Happiness Stud. 18, 177–198. 10.1007/s10902-016-9723-3

[B33] Purcell R. Gwyther K. Rice S. (2019). Mental health in elite athletes: increased awareness requires an early intervention framework to respond to athlete needs. Sports Med. Open 5, 1–8. 10.1186/s40798-019-0220-1 31781988 PMC6883009

[B34] Purdie N. Dudgeon P. Walker R. (2010). Working Together: Aboriginal and Torres Strait Islander Mental Health and Wellbeing Principles and Practice. Commonwealth of Australia.

[B35] Reardon C. L. Hainline B. Aron C. M. Baron D. Baum A. L. Bindra A.. (2019). Mental health in elite athletes: International Olympic Committee consensus statement. Br. J. Sports Med. 53, 667–699. 10.1136/bjsports-2019-100715 31097450

[B36] Rice S. M. Purcell R. De Silva S. Mawren D. McGorry P. D. Parker A. G. (2016). The mental health of elite athletes: a narrative systematic review. Sports Med. 46, 1333–1353. 10.1007/s40279-016-0492-2 26896951 PMC4996886

[B37] Sawyer S. M. Azzopardi P. S. Wickremarathne D. Patton G. (2018). The age of adolescence. Lancet Child Adolesc. Health 2, 223–228. 10.1016/S2352-4642(18)30022-1 30169257

[B38] Seligman M. (2018). PERMA and the building blocks of wellbeing. J. Posit. Psychol. 13, 333–335. 10.1080/17439760.2018.143746635140667

[B39] Seligman M. Adler A. (2018). Positive education. 52–73.

[B40] Seligman M. E. Csikszentmihalyi M. (2014). Positive psychology: an introduction, in Flow and the foundations of positive psychology. Netherlands: Springer. p. 279–298. 10.1007/978-94-017-9088-8_18

[B41] Seligman M. E. P. (2012). Flourish. Sydney, Australia.

[B42] Slemp G. R. Chin T.-C. Kern M. L. Siokou C. Loton D. Oades L. G.. (2017). Positive education in Australia: Practice, measurement, and future directions, in Social and emotional learning in Australia and the Asia-Pacific (Singapore: Springer), 101–122. 10.1007/978-981-10-3394-0_6

[B43] Terare M. Rawsthorne M. (2020). Country is yarning to me: Worldview, health and wellbeing amongst Australian First Nations people. Br. J. Soc. Work. 50, 944–960. 10.1093/bjsw/bcz072

[B44] Tester G. Watkins G. Rouse I. (1999). The sports challenge international programme for identified ‘at risk’children and adolescents: a Singapore study. Asia Pacific J. Public Health. 11, 34–38. 10.1177/101053959901100108 10829826

[B45] Uphill M. Sly D. Swain J. J. (2016). From mental health to mental wealth in athletes: looking back and moving forward. Front. Psychol. 7, 935. 10.3389/fpsyg.2016.00935 27445903 PMC4915134

[B46] Uusiautti S. Leskisenoja E. M. Hyvärinen S. M. (2017). PERMA-based perspectives on sports: designing new ways to support wellbeing in finnish junior ice hockey players. Global J. Human Soc. Sci. 17, 30–39.

[B47] van Agteren J. Iasiello M. Lo L. Bartholomaeus J. Kopsaftis Z. Carey M.. (2021). A systematic review and meta-analysis of psychological interventions to improve mental wellbeing. Nat. Human Behav. 5, 631–652. 10.1038/s41562-021-01093-w 33875837

[B48] Waters L. Loton D. (2019). SEARCH: A meta-framework and review of the field of positive education. Int. J. Appl. Posit. Psychol. 4, 1–46. 10.1007/s41042-019-00017-4

[B49] Waters L. Sun J. Rusk R. Cotton A. Arch A. (2017). Positive education. Wellbeing, Recov. Mental Health. 245, 245–264. 10.1017/9781316339275.021

[B50] Wilson R. L. Waqanaviti K. (2021). navigating first nations social and emotional wellbeing in mainstream mental health services. Nurs. Midwifery Care. 281, 281–306. 10.1017/9781108894166.015

[B51] Xanthopoulos M. S. Benton T. Lewis J. Case J. A. Master C. (2020). Mental health in the young athlete. Curr. Psychiat. Rep. 22, 1–15. 10.1007/s11920-020-01185-w 32954448

