# Supplementary material for: Case Report: An Application of Wellbeing Science for the Development of Adolescent High-Performance Athletes in the Australian Football League
Source: Front Psychol. 2022 Jun 10;13:856241. doi: 10.3389/fpsyg.2022.856241 (PMC9231612; doi:10.3389/fpsyg.2022.856241)
Supplement: Supplementary file 1 [file Table_1.docx]

Supplementary File 1. Table of Mental Fitness Model components

| **Model component** | **Description** | **Theoretical assumptions** | **Activity Examples Designed for the AFL Talent Context^1^** |
| --- | --- | --- | --- |
| *Individual* | The individual athlete is central to the model, with an acknowledgment that each young person’s personality, psychosocial experiences, strengths, and values are unique, and are critical in any effort to promote wellbeing. | It is widely accepted that wellbeing supports should be tailored to an individual’s needs and circumstances (Adler, 2009; Purcell et al., 2022; Rutz, 2009), thus forming the central core of the Model. This element of the model is reinforced with the person-centred principle. | Each individual athlete is assigned a Wellbeing Coordinator who provides tailored one-on-one support, wellbeing planning and goal setting throughout the season.  Individual wellbeing plans are created on a standard THRIVE template:  T: thriving in High Performance;  H: home environment;  R: relationships and social capital;  I: interests and opportunities;  V: vulnerabilities and growth areas;  E: education and employment.  A wellbeing journal provides weekly resources and activities for each training session e.g., weekly game plans; weekly reflections; resilience builders; season in review and a Wellbeing Toolkit. |
|  |  |  |  |
| *Prevent* | The model aims to prevent the development of mental ill-health, through reducing risk factors and promoting protective factors for optimal wellbeing. | The dual continuum of mental wellbeing is reflected through three pillars; Prevent, Support, Thrive (Keyes, Shmotkin, Ryff, & psychology, 2002; Purcell et al., 2022). Wellbeing efforts should aim to both offset risks of mental illness and promote wellbeing. Flourishing is experiencing positive emotion, and to function well in social, psychological and other aspects of life. This model accepts that maintaining wellbeing (flourishing) is possible in the context of mental ill health, and conversely poor wellbeing (languishing) is possible in the absence of mental ill-health. | Facilitated wellbeing conversations and seminars were presented to both male and female athletes on preventative approaches to building wellbeing using an activity-based approach. This included content on the construct of mental fitness and its inclusion in the AFL’s representation of the mental health continuum bespoke to the high performance sport environment.  Female athletes received a seminar on healthy menstruation to empower female athletes and tackle period stigma. ‘The High-Performance Period Webinar’ covers healthy periods, self-monitoring, menstruation impacts on performance and when to reach out for medical advice. |
| *Support* | The model aims to respond early to any signs of mental ill-health, stress and/or languishing by providing individualised support for athletes at their developmental life stage. Supports differ at each stage of the mental wellbeing continuum. |  | Supports for athletes are co-ordinated via regions by locally embedded Wellbeing Coordinators who represent a diverse range of health and education disciplines. All Wellbeing Coordinators are trained in Mental Health Literacy, allied health referral pathways, and youth specific mental health first aid. A community of practice of the Coordinators is maintained throughout the season addressing professional issues e.g., role boundaries and interdisciplinary coordination of responses.  The Wellbeing curriculum is centrally developed, then adapted by the Wellbeing Coordinators to suit each region’s needs. E.g., in regional areas of Victoria Australia, online delivery of wellbeing education to athletes reduces burden on athletes who have excessive travel time around training schedules. |
| *Thrive* | The model aims to promote flourishing through wellbeing literacy and behaviours that are applied to the team sport context. |  | Wellbeing Coordinators implement activities and 1:1 Wellbeing conversations that promote self-determination of youth athletes; facilitate opportunity to contribute meaningfully to the game and others; maximising social capital and peer support; apply growth mindset to individual wellbeing planning; gratitude journaling; and promote engagement with prosocial activities and peers through the collective wellbeing curriculum. |
|  |  |  |  |
| *Promote protective factors* | Mental wellbeing protective factors among youth athletes include positive physical development, protective and prosocial peer relationships; educational attainment; self-esteem; adaptive coping skills; continued engagement in non-athletic endeavours through school, peers or other communities; supportive family environment; and physical and psychological safety in the sport. | The determinants of mental health and wellbeing span biological, physical, environmental, social, economic, behavioural, and other domains (Shokouh et al., 2017; Silva, Loureiro, & Cardoso, 2016). Through promoting protective factors, and through reducing risk factors, the model aims to incorporate public health and health promotion principles to account for the multi-dimensional nature of mental health and wellbeing (Alegría, NeMoyer, Falgàs Bagué, Wang, & Alvarez, 2018). | Football coaches and staff responsible for the development of young athletes are educated on the delicate balance between Wellbeing and performance. Coaches and staff are supported by the Wellbeing Coordinators to create and uphold a psychologically safe environment for young people e.g., address power imbalances; apply safeguarding children principles to all procedures surrounding adult/child interactions; Respectful Relationships training for staff.  Families of players are also educated on the Mental Fitness Model and are encouraged to discuss wellbeing in the home and support the use of the wellbeing journal away from the football club environment. Sharing wellbeing content with the young athlete’s support network (i.e., family) can assist in smooth, regular transitions between home and sporting club. |
| *Reduce risks* | Risk factors for young athletes include:  performance pressures; social media; alcohol use; over-identification with athletic identity (i.e. identity foreclosure); pressures approaching selection; balance of sporting and educational commitments; and recovery from physical injury or sports integrity breaches. |  | Risk factors for abuse of alcohol addressed through ‘just in time teaching’ and psychoeducation sessions delivered in group and 1:1 format at risky times of the season. ‘Problematic drinking conversations’ included safe alcohol choices and the harmful impact of binge use of alcohol on sporting performance, injury recovery, athlete reputation and health.  All AFL staff complete training and adhere to strict child and young people safeguarding standards to ensure a culture of safety that upholds the AFL Safeguarding Children and Young People Policy. |
|  |  |  |  |
| *Positive Emotion* | Positive emotion includes happiness, joy, love, compassion, and other emotions. | These five constructs relate to the PERMA model, developed by Martin Seligman, who proposed that these five elements are major components that contribute toward individual wellbeing (Seligman, 2018). The PERMA model has been associated with other measures of depression, anxiety, and stress (Butler & Kern, 2016; Kern, Waters, Adler, & White, 2015), and has been associated with some healthy developmental milestones in adulthood (O’Connor, Sanson, Toumbourou, Norrish, & Olsson, 2017)(Slemp et al., 2017). | Through the weekly wellbeing journal, athletes are prompted to reflect on positive events during the day or week to cultivate positive emotions. Structured activities are delivered in the wellbeing curriculum that cover completion of gratitude journals, and the practice of daily acts of random kindness. Activities are facilitated within teams and delivered by Wellbeing Coordinators and supported by coaches and the wellbeing journals. |
| *Engagement* | Engagement refers to awareness and presence in the current moment and being fully engaged in the activity at hand. |  | Athletes are supported to acknowledge and explore their unique strengths using the VIA character strengths survey for youth, which is supported by content in the wellbeing journal. Mindfulness is promoted in a variety of activity-based ways including on game days and pre/post training sessions. |
| *Relationships* | Relationships that are positive include interactions that are supporting, loving and where individuals are valued. |  | Team sport fosters relationships and social connection which is capitalised on through activities such as ‘love laps’ of the oval during football training which produce high quality connections between team members and staff. |
| *Meaning* | Meaning refers to holding a purpose, and also includes holding value and worth. |  | The athletes are supported to understand their environment and identify opportunities to support and grow through their individual wellbeing development plans. In addition, athletes are supported to engage with their community through volunteer or fundraising initiatives to experience giving to others and finding a sense of meaning and purpose. |
| *Accomplishment* | Accomplishment is the successful holding and working towards goals. |  | Athletes complete a goal setting exercise whereby they record their goals for both in sport and outside of sport. Athletes also participate in an activity on grit, and how perseverance and passion can support them to achieve their goals. |
|  |  |  |  |
| *Continuum* | Mental health and wellbeing exist on a continuum in that experiences and symptoms may demonstrate mental wellbeing, those at-risk for mental illness, those experiencing sub-threshold symptoms, and those with diagnosable conditions. | Informed by psychiatry’s Staging Model (Hickie, Scott, & McGorry, 2013; McGorry et al., 2007; Scott et al., 2013), the continuum component reflects the understanding that a spectrum of mental illness exists, and that a goal of supporting mental wellbeing is to provide support relative to the experiences of the individual in terms of the illness progression. | In addition to Prevent, Support, Thrive, Mental Fitness was added to the continuum model to acknowledge the unique mental skills required to flourish in the elite sport setting, including resilience and fortitude. A working definition of mental fitness identified through Delphi methods found mental fitness to be the capacity to flexibly use resources and skills to adapt to challenges, and enabling thriving (Robinson, Oades, & Caputi, 2015). Athletes receive education on the mental health continuum which increased mental health literacy and reduces stigma about accessing help at any stage of the continuum. Additional resources are provided to them in their wellbeing journal. This includes a detailed How to Seek help guide which presents various options for support available to athletes at each section of the continuum. |
| *Life cycle* | The life cycle component reflects the acknowledgment that mental wellbeing supports are required to be developmentally and age appropriate to best support the needs of young athletes. The mental wellbeing experiences, pedagogical approaches and thus activities to support wellbeing are tailored to late adolescence. | The model adopts a developmental perspective in that the curriculum components, the skills required in support personnel, and other wellbeing activities, are age and developmentally appropriate to meet the needs of the young athletes in late adolescence (Barnes & medicine, 1998; Thapar & Riglin, 2020). | Activities are developmentally appropriate, for example utilising the Values in Action character strengths survey for youth and introducing parents and guardians to the model and program to optimize continued support in the home for the young people.  The young men’s and women’s programs were time specific relative to the educational commitments of the athletes (e.g., respectful of final year schooling exams) and the specific timing of the AFL and AFLW draft and competition timing. |
| *Person Centred* | The model incorporates person centred approach in assuming each individual will have experiences and meaning of mental wellbeing. Activities and supports that are informed by this model hold that that each individual athlete has unique strengths and abilities which are respected and empowered. Further, this model aims to incorporate the views and experiences of young people alongside best practice principles to develop mental wellbeing activities, to ensure there is a partnership in mental wellbeing support. | Person-centred approaches adopt the principles that all people are treated with respect, care and supports that are co-ordinated, and centred on the individual needs, and recognise and focus on building and developing unique individual strengths and abilities (Mead, Bower, & medicine, 2000; Salvador-Carulla, Mezzich, & Sciences, 2012). | Individual wellbeing development plans (THRIVE) are developed with each of the talent pathway athletes. This plan includes a comprehensive understanding of the athletes’ non-athletic endeavours, levels of support, areas of vulnerability and goals for development. The outcome is to identify the athletes’ personal strengths, possible challenges, and opportunities to support the athlete.  Evaluation activities include participatory methods to revise the model components, in particular how the appropriateness of the language used throughout wellbeing activities to ensure relevance to the athletes, and to the AFL sporting system. |
| *Shared responsibility* | This model assumes that the mental health and wellbeing outcomes of individuals are a shared responsibility across the sporting ecosystem. Everyone has a role to play in the ecosystem including athletes, coaches, support staff, families, and other personnel. | The social ecological model recognises the systems surrounding individuals are important interacting factors which impact outcomes (Bronfenbrenner, 1986; Purcell et al., 2022). As such, whilst there are some discrete activities in the wellbeing model (e.g., wellbeing curriculum) it is expected that the practice of wellbeing occurs through the young person’s engagement with the sporting ecosystem, and that there is a shared responsibility for mental wellbeing throughout their experiences. | The wellbeing coordinator’s role is to facilitate the development and implementation of wellbeing strategies and manage the delivery of the wellbeing curriculum. They provided individual support and guidance to drive athletes to pursue good mental health and wellbeing, as well as linking the sporting club (e.g., coaches and other support staff) with the larger AFL system (e.g., the Mental Health and Wellbeing team). In addition, the wellbeing Coordinator worked closely with the coaching and management staff to ensure that wellbeing was a shared conversation amongst the people responsible for the development of young athletes. Prior to the wellbeing program commencing coaches and talent managers received education in the mental fitness model, developmental stages of young people and safeguarding children. This allowed for the collective level of knowledge in the sporting environment to be raised which in turn supported the work of the wellbeing coordinators and ultimately the young athletes. |

^1^Note: Some activities are not exclusive to individual components, thus reflecting the cyclical and interactive nature of the Mental Fitness Model. Further, these examples are not an exhaustive list of activities

**References**

Adler, R. H. J. J. o. p. r. (2009). Engel's biopsychosocial model is still relevant today. *67*(6), 607-611.

Alegría, M., NeMoyer, A., Falgàs Bagué, I., Wang, Y., & Alvarez, K. J. C. p. r. (2018). Social determinants of mental health: where we are and where we need to go. *20*(11), 1-13.

Barnes, J. J. P., health, & medicine. (1998). Mental health promotion: A developmental perspective. *3*(1), 55-69.

Bronfenbrenner, U. J. D. p. (1986). Ecology of the family as a context for human development: Research perspectives. *22*(6), 723.

Butler, J., & Kern, M. L. J. I. J. o. W. (2016). The PERMA-Profiler: A brief multidimensional measure of flourishing. *6*(3).

Hickie, I. B., Scott, J., & McGorry, P. D. J. T. M. J. o. A. (2013). Clinical staging for mental disorders: a new development in diagnostic practice in mental health. *198*(9), 461-462.

Kern, M. L., Waters, L. E., Adler, A., & White, M. A. J. T. j. o. p. p. (2015). A multidimensional approach to measuring wellbeing in students: Application of the PERMA framework. *10*(3), 262-271.

Keyes, C. L., Shmotkin, D., Ryff, C. D. J. J. o. p., & psychology, s. (2002). Optimizing wellbeing: the empirical encounter of two traditions. *82*(6), 1007.

McGorry, P. D., Purcell, R., Hickie, I. B., Yung, A. R., Pantelis, C., & Jackson, H. J. J. M. J. o. A. (2007). Clinical staging: a heuristic model for psychiatry and youth mental health. *187*(S7), S40-S42.

Mead, N., Bower, P. J. S. s., & medicine. (2000). Patient-centredness: a conceptual framework and review of the empirical literature. *51*(7), 1087-1110.

O’Connor, M., Sanson, A. V., Toumbourou, J. W., Norrish, J., & Olsson, C. A. J. J. o. H. S. (2017). Does positive mental health in adolescence longitudinally predict healthy transitions in young adulthood? *, 18*(1), 177-198.

Purcell, R., Pilkington, V., Carberry, S., Reid, D., Gwyther, K., Hall, K., . . . Rice, S. J. F. i. P. (2022). An Evidence-Informed Framework to Promote Mental Wellbeing in Elite Sport. *13*, 780359-780359.

Robinson, P., Oades, L., & Caputi, P. J. I. J. o. W. (2015). Conceptualising and measuring mental fitness: A Delphi study. *5*(1).

Rutz, W. (2009). Positive Health and Health Promotion: The WPA Institutional Programme of Psychiatry for the Person in a European Public Health Perspective. In *Psychiatric Diagnosis*.

Salvador-Carulla, L., Mezzich, J. J. E., & Sciences, P. (2012). Person-centred medicine and mental health. *21*(2), 131-137.

Scott, J., Leboyer, M., Hickie, I., Berk, M., Kapczinski, F., Frank, E., . . . McGorry, P. J. T. B. J. o. P. (2013). Clinical staging in psychiatry: a cross-cutting model of diagnosis with heuristic and practical value. *202*(4), 243-245.

Seligman, M. J. T. J. o. P. P. (2018). PERMA and the building blocks of wellbeing. *13*(4), 333-335.

Shokouh, S. M. H., Mohammad, A., Emamgholipour, S., Rashidian, A., Montazeri, A., & Zaboli, R. J. I. j. o. p. h. (2017). Conceptual models of social determinants of health: a narrative review. *46*(4), 435.

Silva, M., Loureiro, A., & Cardoso, G. J. T. E. J. o. P. (2016). Social determinants of mental health: a review of the evidence. *30*(4), 259-292.

Slemp, G. R., Chin, T.-C., Kern, M. L., Siokou, C., Loton, D., Oades, L. G., . . . Waters, L. (2017). Positive education in Australia: Practice, measurement, and future directions. In *Social and emotional learning in Australia and the Asia-Pacific* (pp. 101-122): Springer.

Thapar, A., & Riglin, L. J. M. p. (2020). The importance of a developmental perspective in Psychiatry: what do recent genetic-epidemiological findings show? *, 25*(8), 1631-1639.
